# Oil of Turpentine in Post-Partum Hemorrhage

**Published:** 1898-06

**Authors:** S. H. Stout

**Affiliations:** Dallas, Texas


### TEXAS MEDICAL JOURNAL.

ESTABLISHED JULY, 1885.

PUBLISHED MONTHLY.—SUBSCRIPTION $2.00 A YEAR.

VOL. XIII. AUSTIN, JUNE, 1898. No. 12.

### Original Contributions.

For the Texas Medical Journal.

### Oil of Turpentine in Post-Partum Hemorrhage.

BY S. H. STOUT, A. M., M. D., L. L. D., DALLAS, TEXAS.

Long before 1 became a student of medicine, and before ergot of rye had obtained a place in the pharmacopœa, many practitioners of medicine were known to have used teaspoonful doses of oil of turpentine given in sweetened water, mucilage or starch water, to check post-partum uterine hemorrhage. In my own practice I have given it with gratifying results. While a resident of Eastland county, Texas, my use of it seemed a novelty to the midwives and the physicians residing there. Some notoriously happy results of its use by myself are among the traditions of my practice in that county, where I resided eleven years.

A recent enquiry by a physician now living in that county as to my estimate of the value of oil of turpentine in uterine hemorrhages, and the extent and limitations of its use by me, has suggested the propriety of preparing this paper and sending it to the TEXAS MEDICAL JOURNAL for publication.

It is appropriate now to state that I have never used it as a means of exciting uterine contractions save in cases of full term; and that for obvious reasons, 1 think it contra-indicated in cases of menorrhagia, metrorrhagia, in abortions or miscarriages (excepting in miscarriages near the full term and after the womb has been emptied of its contents), and in hemorrhages occurring during the changes preceding the menopause, except in certain cases hereafter alluded to.

Parturition at full term is physiological. Abortions and miscarriages are pathological, and declare the existence of previous systemic disease of the mother or her genital organs, or in some part or parts of the ovum, or the effect of mechanical injury, accidental or criminal; or the result of scientifically justifiable surgical interference,—also the injection of oxytocics, criminal or otherwise.

All of us know that the saving of a mother at full term from death consequent upon post-partum hemorrhage is due to the prompt contraction of the muscular fibers of the uterus after the detachment of the placenta. Whatever stimulates or promotes that contraction is scientifically indicated. Since the introduction of ergot in obstetric practice, the use of oil of turpentine as a means of insuring uterine contraction has almost lapsed from the memory of medical men of the present day. But he or she who essays to conduct a mother through the travails of childbirth ought to be resourceful, and should keep the memory refreshed with every practical and rational means of saving the mother from death when her organism is performing physiological functions, and after the birth of her child is instinctively engaged in the physiological processes that produce such changes as restore her organism to the condition of that of an healthy non-pregnant woman.

To the right understanding of the modus operandi of oil of turpentine as a prompt and certain stimulator of uterine contraction after the emptying of the womb at full term, it is proper to here rehearse its physiological action. Given in teaspoonful doses or larger, and even sometimes in less doses, it is a general stimulant, and its volatility is such that it penetrates the tissues of the body without having to do so by aid of the vital process of absorption. It has a decided local tendency towards the urinary organs, stimulating the kidneys and the whole urinary track. It so promptly reaches the urinary system that it may give odor to the urine in a very short time;—some have reported that its odor has been detected in the urine in less than a minute after its ingestion by the mouth. In large doses it intoxicates, causing the patient to stagger and often to complain of vertigo. Strangury and bloody urine often follow its ingestion in large doses. Oil of turpentine, like all the volatile oils, is antiseptic, and for its betraying penetrating order would be a valuable addition to the aromatic wine so much used fifty years or more ago in the dressing of syphilitic sores, and to Listerine so much used at the present time.

Oil of turpentine, when we consider its physiological action, is therefore rationally indicated in the shock that often follows childbirth, and the non-contraction of the muscular fibers of the emptied womb. Its specific tendency to stimulate the urinary organs and the mucous lining of the urinary track, and organs in their vicinity, rationally indicate its use as a stimulant of the genital track and per consequence of the uterine fibers.

In every case where I have given it in post-partum hemorrhage my patient has never suffered from strangury or voided bloody urine. This I account for by considering that at full term the instinct of the woman's organism is so actively engaged in the physiological work of passing out through the genital and urinary tract effete materials that it also eliminates the oil of turpentine so rapidly that the medicine is not retained long enough to produce strangury or bloody urine.

I have never prescribed oil of turpentine to moderate the fearful uterine hemorrhages that sometimes afflict women during the efforts of the instinct of her organism to complete the menopause, having always secured satisfactory moderation of them by rest, the administration of elixir vitriol in ten to fifteen drops, largely diluted, every three or four hours, ergot, etc. But in extreme cases, in which the womb is of normal temperature and the patient much exhausted, after other remedies seem to be inefficient, I would not hesitate to use oil of turpentine in heroic doses. For during the effort to bring about the menopause the woman's organism is engaged like in childbirth at full term in the performing of those physiological functions which we may expect would promote the elimination of the medicine before it has been long enough retained to produce strangury or bloody urine.

Owing to the pathological state of the genital organs in miscarriages and abortions, I think the medicine in heroic doses in general is contra-indicated.

In a remote rural district I was once well acquainted with a practitioner who was an empirical routinist, who gave teaspoonful doses of oil of turpentine to his patients (and he had a numerous clientelle) upon the conclusion of every case of full-term childbirth, whether it was a normal one or otherwise. He told me that he had never encountered strangury or bloody urine as a consequence thereof. He also stated that after giving it in such cases he was never recalled to the patient's bedside on account of the recurring of hemorrhage. This statement I listened to *cum grano salis.*

